# Supplementary material for: Single-cell transcriptomics-informed induced pluripotent stem cells differentiation to tenogenic lineage
Source: eLife. 2026 May 21;12:RP89652. doi: 10.7554/eLife.89652 (PMC13193713; doi:10.7554/eLife.89652)
Supplement: Supplementary file 1. [file elife-89652-supp1.docx]

**Supplementary File**

**Supplementary file 1:** Normalized cell counts (expressed as %) per cluster following WNTi treatment, shown for the two cell lines, 007i and 83i.

| **Cluster** | **Annotation** | **Markers** | **WNTi-007i**  **(% Normalized counts)** | **WNTi-83**  **(% Normalized counts)** |
| --- | --- | --- | --- | --- |
| **0** | SYN | MKX+TNMD+DCN+BGN | 66.85562 | 78.49919 |
| **1** | Mesoderm (Mes) | MIXL+TBXT+MSGN1+DLL1+DLL3+ | 3.40124 | 1.678803 |
| **2** | iPSC-1 | OCT4+,NANOG+LIN28A+SOX2+ | 3.418955 | 0.667476 |
| **3** | iPSC-2 | OCT4+,NANOG+LIN28A+SOX2+ | 2.710363 | 0.606796 |
| **4** | iPSC-3 | OCT4+,NANOG+LIN28A+SOX2+ | 3.790965 | 0.52589 |
| **5** | NMP/NC | TBXT/TWIST1/SP5/SNAI2 | 0.318866 | 0.101133 |
| **6** | iPSC-4 | OCT4+LIN28A+SOX2+ | 2.409212 | 2.04288 |
| **7** | iPSC-5 | OCT4+NANOG+LIN28A+SOX2+ | 3.525244 | 8.555825 |
| **8** | Neural crest | NTRK2+SOX4+SOX11+ | 4.69442 | 7.038835 |
| **9** | Fibrocartilage (FC) | COL2A1+SOX9+FN1+BGN+COL1A1 | 8.733392 | 0.262945 |
| **10** | Neural Lineage (NL) | SOX2+DCX+MAP2+UNCX+SOX4+ | 0.053144 | 0 |
| **11** | iPSC-6 | OCT4+NANOG+LIN28A+SOX2+ | 0.088574 | 0.020227 |
